# Interleukin-26 Expression in Inflammatory Bowel Disease and Its Immunoregulatory Effects on Macrophages

**DOI:** 10.3389/fmed.2022.797135

**Published:** 2022-04-06

**Authors:** Dongjuan Song, Lijie Lai, Juntao Lu, Jinlu Tong, Zhihua Ran

**Affiliations:** Division of Gastroenterology and Hepatology, Key Laboratory of Gastroenterology and Hepatology, Ministry of Health, Inflammatory Bowel Disease Research Center, Renji Hospital, School of Medicine, Shanghai Jiao Tong University, Shanghai Institute of Digestive Disease, Shanghai, China

**Keywords:** inflammatory bowel disease, IL-26, disease activity, macrophages, transcriptome analyses

## Abstract

**Background and Aim:**

Interleukin-26 (IL-26) has been implicated in several chronic inflammatory diseases. However, its role in inflammatory bowel disease (IBD) remains to be elucidated. We aimed to investigate IL-26 expression in IBD and its immunoregulatory effects on macrophages.

**Methods:**

We assessed IL-26 expression in the intestinal mucosa and blood samples of IBD patients and healthy controls (HC). The associations between the clinical characteristics of IBD and IL-26 expression levels in serum and peripheral blood mononuclear cells (PBMCs) were investigated. In addition, the transcriptional changes in THP-1 macrophages exposed to IL-26 were determined by RNA sequencing and validated with qRT-PCR, ELISA and western blots.

**Results:**

Compared with HC, in IBD patients, IL-26 expression levels were elevated in the inflamed intestinal mucosa, and reduced in serum and PBMCs. IL-26 mRNA levels in PBMCs, but not serum IL-26 levels, were inversely correlated with disease activity in IBD. Furthermore, IL-26 mRNA levels in PBMCs were significantly lower in patients with complicated Crohn’s disease. A total of 1,303 differentially expressed protein-coding genes were identified between untreated and IL-26-treated macrophages. The up-regulated genes showed enrichment in some inflammatory and immune-related processes and pathways. Additionally, GSEA showed that neutrophil, monocyte, and lymphocyte chemotaxis was significantly enriched in IL-26-treated macrophages. Further validation revealed that IL-26 promotes the secretion of multiple inflammatory cytokines and chemokines and upregulates the expression of adhesion molecules, MMP-8, and MMP-9 while inhibiting MMP-1 in macrophages.

**Conclusion:**

Compared with HC, in IBD patients, IL-26 levels were elevated in the inflamed intestinal mucosa, and reduced in the peripheral blood. The transcriptional changes in macrophages exposed to IL-26 suggest that IL-26 may amplify the aberrant immune response in IBD by activating macrophages.

## Introduction

Inflammatory bowel disease (IBD), including ulcerative colitis (UC) and Crohn’s disease (CD), is associated with inappropriate immune system activation and excessive inflammatory cytokine production. The lack of a pro- vs. anti-inflammatory cytokine balance in IBD leads to tissue destruction and disease progression (1). Some anti-cytokine agents, such as TNF-α inhibitors and antibodies that targets IL-12/IL-23, have been proven to be effective against IBD (2). Therefore, novel cytokine-directed therapies may be promising for IBD treatment.

Interleukin-26 (IL-26) is a member of the IL-10 cytokine family, which also includes IL-10, IL-19, IL-20, IL-22, IL-24, IL-28A, IL-28B, and IL-29. These cytokines signal through common or different receptor complexes consisting of α-receptor subunits (IL-10RA, IL-20RA, IL-22R, or IL-28R) and β-receptor subunits (IL-10RB or IL-20RB) (3). Several studies have indicated that IL-26 not only acts as an inflammatory mediator but also participates in antiviral immunity and antimicrobial defense (4). Elevations in IL-26 are noted in individuals with rheumatoid arthritis, Behçet’s disease (BD), atopic dermatitis (AD), psoriasis, asthma, and IBD, implicating this cytokine in the development of chronic inflammatory and autoimmune disorders (5–10). Macrophages, T helper 17 cells (Th17 cells), CD4^+^ T cells, CD8^+^ T cells, NKp30^+^ γδ T cells and natural killer cells in the intestinal mucosa of IBD patients can produce IL-26 (5, 11–13). IL-26 enhances the production and expression of pro-inflammatory cytokines and chemokines in human monocytes, epidermal keratinocytes, intestinal epithelial cells and colonic subepithelial myofibroblasts (SEMFs) (5, 6, 8, 11). In particular, IL-26 promotes Th17 cell production (6, 9), and these cells in turn produce IL-26, leading to a detrimental amplification loop that contributes to Th17-associated inflammation. Importantly, compared with control mice, transgenic mice expressing human IL-26 exhibit exacerbated skin inflammation as well as increased vascularization and infiltration by neutrophils and CD4^+^ T cells, and these effects are attenuated by anti-IL-26 monoclonal antibody administration (14, 15). Additionally, IL-26 administration aggravates oxazolone-induced AD in mice via the promotion of the Th2 and Th17 response and increases mortality in mouse models of sepsis, accompanied by increased infiltration of neutrophils into the peritoneal cavity (8, 16). In contrast, a previous study has shown that IL-26 inhibits IL-8, IL-1β, TNF-α, and GM-CSF secretion from bronchial epithelial cells (17). Importantly, human IL-26 transgenic mice show reduced susceptibility to the acute colitis caused by dextran sodium sulfate (DSS) (12). Such evidence suggests that the immunoregulatory effects of IL-26 may be pleiotropic and cell-dependent.

Macrophages are among the most abundant immune cells in the gut, playing indispensable roles in the maintenance of intestinal homeostasis and host defense (18). Macrophages exhibit high plasticity and can modify their phenotypes and physiology based on environmental signals. Different subgroups of macrophages perform different functions—host defense, wound healing, and immune regulation (19). Macrophage dysregulation is involved in the pathology of IBD (20). Macrophages can release multiple pro-inflammatory cytokines and chemokines, which are associated with tissue damage in IBD (21). Additionally, macrophages can produce matrix metalloproteinases (MMPs), which contribute to the progression of IBD through their role in extracellular matrix (ECM) remodeling (22). Unraveling the immunoregulatory effects of IL-26 on macrophages may provide critical insights into the roles of IL-26 in IBD. IL-26 has been reported to polarize macrophages into the M1 phenotype and enhance the expression of CCL20, CXCL8, and CXCL2 mRNA as well as the secretion of TNF-α in THP-1 macrophages (23, 24). However, to date, a comprehensive transcriptomic analysis of IL-26-exposed macrophages has not been previously reported.

Previous studies have reported increased IL-26 levels in inflamed intestinal mucosa of IBD patients and elevated serum IL-26 levels in individuals with CD as compared with healthy controls (HC) (5, 11). However, no previous study has explored IL-26 mRNA levels in PBMCs of IBD patients and serum IL-26 levels in UC as well as their associations with clinical characteristics. Thus, we investigated IL-26 expression in the intestinal mucosa, serum and PBMCs from patients with IBD and HC. Further, we examined the association of serum IL-26 levels as well as IL-26 mRNA expression in PBMCs with the clinical features of UC and CD, including disease activity and phenotypes. Finally, in order to gain additional insights into the immunoregulatory effects of IL-26 on macrophages, we performed RNA sequencing and validated the results using qRT-PCR, ELISA, and western blots.

## Materials and Methods

### Patients

Samples of intestinal mucosa and blood were collected from individuals with IBD and HC after obtaining signed informed consent. All participants were enrolled from Renji Hospital, School of Medicine, Shanghai Jiao Tong University, and the study was approved by the Medical Ethics Committee of Renji Hospital, School of Medicine, Shanghai Jiao Tong University.

The histological activity of IBD patients was classified as histologic normalization, quiescence, and active disease, as described earlier (25). Endoscopic remission was defined as the absence of any ulceration for CD and Mayo endoscopic subscore ≤ 1 for UC (26). For qRT-PCR analysis, paired macroscopically inflamed and uninflamed biopsies were obtained from the rectum and colon for UC patients and from the rectum, colon, caecum, ileocecal valve, and terminal ileum for CD patients. Normal colonic biopsies were obtained from HC. Baseline characteristics of participants at intestinal biopsy are shown in Supplementary Table 1. In addition, formalin-fixed paraffin-embedded tissue sections were subjected to immunohistochemical analysis (Supplementary Table 2).

We centrifuged blood samples and stored serum at –80°C until IL-26 quantitation. Human PBMCs were isolated from freshly obtained blood using density gradient centrifugation with Ficoll-Paque Plus (GE Healthcare, North Richland Hills, TX, United States). The disease activity of UC and CD was determined according to the modified Truelove and Witts activity index and Harvey-Bradshaw index, respectively (27, 28). Laboratory values for white blood cell (WBC) counts, levels of hemoglobin (Hb), C-reactive protein (CRP), erythrocyte sedimentation rate (ESR), and prealbumin as well as serum levels of cytokines such as IL-1β, IL-2, IL-4, IL-5, IL-6, IL-8, IL-10, IL-12p70, IL-17A, TNF-α, IFN-α, and INF-γ were obtained from electronic medical records. Clinical characteristics of all enrolled individuals at blood sample collection are described in Supplementary Table 3.

### Serum Interleukin-26 Quantification

Serum IL-26 levels were detected using a human IL-26 ELISA kit (Cusabio Biotech, Wuhan, China).

### RNA Isolation and Quantitative Real-Time Polymerase Chain Reaction

We extracted total RNA using the RNAiso Plus kit (Takara, Japan) and then reverse transcribed it (1 μg RNA) with the PrimeScript™ RT reagent Kit (Takara). We performed real-time polymerase chain reaction (PCR) with the TB Green^®^ Premix Ex Taq™ II kit (Takara), using the 2^–ΔΔ^*Ct* method for quantifying relative expression. GAPDH and ACTB were chosen as internal controls. Primers are shown in Supplementary Table 4.

### Immunohistochemistry

Briefly, 4-μm tissue sections were first deparaffinized and rehydrated and then heated for antigen retrieval after quenching endogenous peroxidase activity with 3%H_2_O_2_. The sections were then incubated with an anti-IL-26 antibody (Abcam, Cambridge, United Kingdom) at 4°C overnight after blocking with non-immune goat serum for 30 min. Phosphate-buffered saline (PBS) was used to wash sections, following which they were incubated with a horseradish peroxidase (HRP)-conjugated secondary antibody (Changdao, Shanghai, China) for 30 min at room temperature. 3,3′-diaminobenzidine (DAB, Maixin-Bio, Guangzhou, China) was used to visualize IL-26 expression, and hematoxylin was used for counterstaining the sections. Immunohistochemical results were analyzed using the Image-Pro Plus 6.0 software (Media Cybernetics, Rockville, MD). The level of immunoreactivity was expressed as the mean optical density (integrated optical density/area).

### Cell Culture and Treatment

THP-1 monocytes (ATCC, Manassas, VA, United States) were cultured in RPMI 1,640 medium (Gibco, United States) containing 10% fetal bovine serum (FBS). Phorbol12-myristate 13-acetate (PMA, 50 ng/ml, Sigma-Aldrich, United States) was added for 24 h to induce the differentiation of THP-1 cells into macrophages. Then, THP-1 macrophages (M0 phenotype) underwent IL-26 treatment (0.28 μM, R&D Systems, Minneapolis, MN, United States) for 24 h.

### RNA-Sequencing and Analysis

The constructed sequencing library was sequenced using an Illumina NovaSeq 6000 system. The expression of transcripts was calculated as fragments per kilobase of exon model per million mapped reads (FPKM). Protein-coding genes were used for subsequent analysis. Differentially expressed genes were identified based on the following criteria: fold change ≥ 2 or ≤ 0.5 and FDR < 0.05.

Gene Ontology (GO) enrichment analysis and Kyoto Encyclopedia of Genes and Genomes (KEGG) pathway analysis were performed using the OmicStudio tools at https://www.omicstudio.cn/tool. FDR values < 0.05 indicated significant enrichment. The gene sets (hallmark gene sets, KEGG gene sets, and GO biological processes gene sets) were obtained from the Molecular Signatures Database (MsigDB)^1^ for GSEA enrichment analysis (4.1.0). Gene sets with |NES| > 1 and FDR *q*-value < 0.05 were considered significant.

### Enzyme-Linked Immunosorbent Assay

After IL-26 treatment for 24 h, the concentration of cytokines and chemokines in the cell culture supernatants of THP-1 macrophages was measured with enzyme-linked immunosorbent assay (ELISA) according to manufacturer’s instructions. ELISA kits for the detection of human TNF-α, IL-8, IL-1β, CCL2, CCL4, CCL8, CCL20, CXCL1, CXCL2, and CXCL10 were purchased from Cusabio Biotech. Human CXCL3 and CXCL11 ELISA kits were obtained from MultiSciences (Hangzhou, China). Human CCL3 ELISA kits were purchased from Absin (Shanghai, China).

### Protein Isolation and Western Blot Analysis

Cell lysates were obtained after the addition of radioimmunoprecipitation assay lysis buffer (Beyotime, Shanghai, China) containing a cocktail of protease inhibitors (KangChen, China) to the cells. Proteins were quantified using a bicinchoninic acid Protein Assay Kit (Thermo Fisher Scientific, Lafayette, United States). Equal amounts of protein were separated using 10% SDS-PAGE and transferred to PVDF membranes (Bio-Rad, Hercules, CA). Membranes were blocked in 5% skim milk for 1 h and subsequently incubated with primary antibodies against MMP-9 (Cell Signaling Technology, Boston, MA), MMP-1 (Proteintech Group, United States), MMP-8 (Abcam), vascular cell adhesion molecule-1 (VCAM-1) (Abcam), intercellular adhesion molecule 1 (ICAM-1) (Abcam), and GAPDH (KangChen, China) at 4°C overnight. Membranes were washed thrice with TBS-T, and then incubated with HRP-conjugated secondary antibodies at room temperature for 1 h. Protein signals were detected using an ECL detection system.

### Statistical Analysis

Data were presented as mean ± standard deviation (SD) and analyzed using GraphPad Prism 6.0 or SPSS software. For data with normal distribution, comparisons between two groups were performed using paired or unpaired Student’s *t*-tests, and multigroup comparisons were conducted using one-way analysis of variance (ANOVA). Non-parametric tests were employed for non-normally distributed continuous variables. Pearson’s and Spearman’s correlation analyses were used for data with normal distribution and non-normal distribution, respectively. *P*-values lower than 0.05 indicated statistical significance.

## Results

### Interleukin-26 Expression in the Intestinal Mucosa of Individuals With Inflammatory Bowel Disease

**FIGURE 1:**
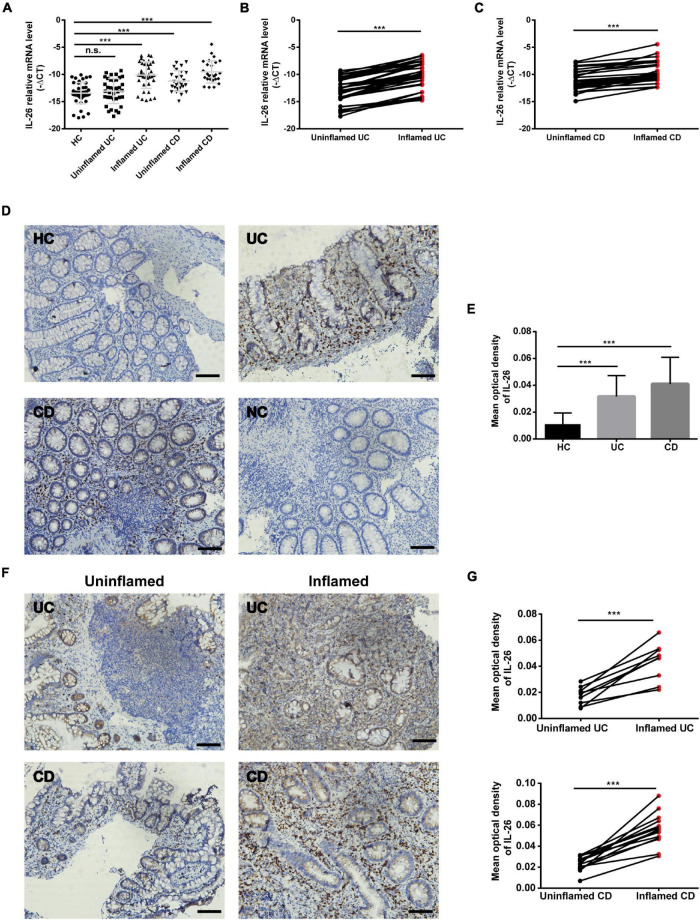
IL-26 expression is increased in the inflamed intestinal mucosa of IBD patients. **(A)** IL-26 mRNA levels in inflamed and uninflamed intestinal mucosa from UC patients (*n* = 37) and CD patients (*n* = 26), and normal intestinal mucosa from healthy controls (HC) (*n* = 45). **(B,C)** IL-26 mRNA levels in paired inflamed and uninflamed intestinal mucosa from patients with UC (*n* = 37) **(B)** and patients with CD (*n* = 26) **(C)**. **(D)** Immunohistochemical staining for IL-26 in intestinal mucosa from patients with UC and CD, and HC. Negative control (NC): omission of the primary antibody. Scale bar, 100 μm. **(E)** The semi-quantitative analysis of immunohistochemistry staining of IL-26 in intestinal mucosa from patients with UC (*n* = 22) and CD (*n* = 28), and HC (*n* = 23). **(F)** Immunohistochemical staining for IL-26 in paired inflamed and uninflamed intestinal mucosa from the same UC and CD patients. Scale bar, 100 μm. **(G)** The semi-quantitative analysis of immunohistochemistry staining of IL-26 in paired inflamed and uninflamed intestinal mucosa from the same UC and CD patients. ***P < 0.001. n.s., not significant.

We analyzed IL-26 RNA transcript and protein levels in the intestinal mucosa of UC patients, CD patients, and HC using qRT-PCR and immunochemistry, respectively. IL-26 mRNA levels were significantly upregulated in inflamed intestinal mucosa from patients with UC and CD when compared with HC (Figure 1A). These levels were also enhanced in inflamed intestinal mucosa compared with uninvolved intestinal mucosa from the same patients with UC or CD (Figures 1B,C). In line with this, there were more IL-26-expressing cells in the intestinal mucosa of patients with UC and CD than in HC (Figures 1D,E). Furthermore, IL-26 protein expression was more pronounced in inflamed than in non-inflamed intestinal mucosa from the same UC or CD patients (Figures 1F,G). These findings illustrated that IL-26 expression is upregulated in the inflamed intestinal mucosa of patients with IBD.

### Interleukin-26 Expression Levels in Serum and Peripheral Blood Mononuclear Cells of Inflammatory Bowel Disease Patients and Their Association With Clinical Characteristics

**FIGURE 2:**
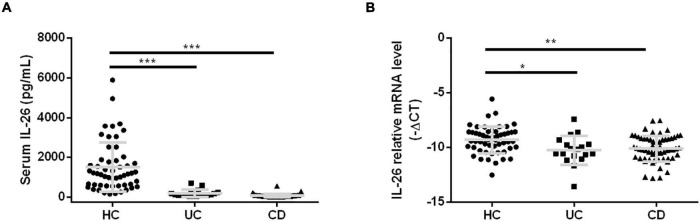
IL-26 expression levels in serum and PBMCs were reduced in IBD patients. IL-26 expression levels in serum **(A)** and PBMCs **(B)** in UC patients, CD patients and healthy controls (HC). *P < 0.05, **P < 0.01, ***P < 0.001.

Previous studies have reported disease severity-related serum IL-26 elevations in cases of systemic lupus erythematosus, BD, and sepsis (7, 16, 29). We aimed to explore IL-26 expression levels in serum and PBMCs in cases of UC and CD and their association with clinical characteristics. To our surprise, we found lower serum IL-26 concentrations in UC and CD patients than in HC (Figure 2A), and a similar trend was also observed for IL-26 mRNA expression in PBMCs (Figure 2B). Subsequently, we compared IL-26 expression levels in serum and PBMCs among IBD patients with different clinical parameters. Regarding disease location, IL-26 expression levels in serum and PBMCs were comparable between CD patients with terminal ileum involvement (L1 ± L4) and colonic involvement (L2/L3 ± L4) (Supplementary Figures 1A,B), and between CD patients with and without upper gastrointestinal involvement (Supplementary Figures 1C,D). However, we could not compare IL-26 levels among UC patients due to the small sample size. Interestingly, IL-26 mRNA levels in PBMCs were observed to be lower in cases of complicated CD (stricturing and penetrating) than in cases of inflammatory disease, although no such trend was noted for serum IL-26 levels (Supplementary Figures 1E,F). These results indicated that IL-26 expression levels in serum and PBMCs were reduced in cases of UC and CD compared to HC and were unaffected by disease location in CD. Furthermore, IL-26 mRNA expression was lower in PBMCs from CD patients with stricturing and penetrating phenotypes than in those with inflammatory phenotype.

**FIGURE 3:**
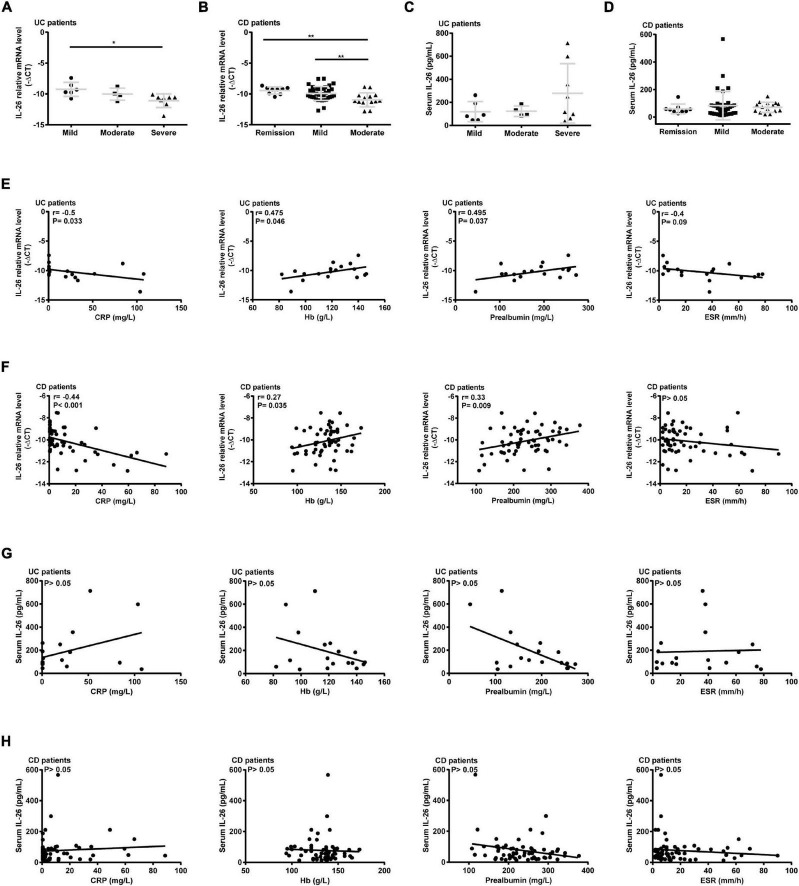
Correlation of IL-26 levels in serum and PBMCs with disease activity in patients with UC and CD. **(A,B)** IL-26 mRNA levels in PBMCs of UC **(A)** and CD patients **(B)** with different disease activity levels. **(C,D)** Serum IL-26 concentration in UC **(C)** and CD patients **(D)** with different disease activity levels. **(E,F)** Correlation of IL-26 mRNA levels in PBMCs with levels of CRP, hemoglobin (Hb), prealbumin, and ESR in cases of UC **(E)** and CD **(F)**. **(G,H)** Relationship of serum IL-26 levels with levels of CRP, Hb, prealbumin, and ESR in UC **(G)** and CD **(H)** patients. *P < 0.05, **P < 0.01.

In terms of disease activity, IL-26 expression in PBMCs was significantly lower in severely active UC than in mildly active UC (Figure 3A) and in moderately active CD than in remitted and mildly active CD (Figure 3B). In contrast, disease activity did not affect serum IL-26 levels in UC and CD patients (Figures 3C,D). In addition, IL-26 expression in PBMCs from UC patients was negatively correlated with the CRP levels and positively correlated with Hb and prealbumin levels (*P* < 0.05). These levels also tended to show a negative correlation with ESR, although the correlation did not reach statistical significance (Figure 3E). IL-26 expression in PBMCs from CD patients also showed a negative correlation with CRP levels and a positive correlation with Hb and prealbumin levels (*P* < 0.05), but showed no correlation with ESR (Figure 3F). In contrast, serum IL-26 levels were not associated with levels of CRP, Hb, prealbumin, and ESR in either UC or CD (Figures 3G,H). These results illustrated that IL-26 mRNA levels in PBMCs may show a negative correlation with disease activity in both UC and CD, although serum IL-26 levels may not show any correlation with disease activity.

We further explored the association of serum IL-26 levels with those of other cytokines in UC and CD (Table 1). Serum IL-26 levels showed a positive correlation with serum IL-6 levels in UC and CD (*P* < 0.05) and with serum IL-8 levels in CD (*P* < 0.05). A tendency toward a positive correlation with serum IL-8 levels was noted in UC.

**TABLE 1:** Correlation analysis between serum levels of IL-26 and other cytokines in IBD.

Cytokines	UC patients		CD patients	
	Correlation coefficient	*P*-value	Correlation coefficient	*P*-value
IL-1β	0.14	0.61	0.036	0.79
IL-2	–0.08	0.77	–0.17	0.2
IL-4	–0.17	0.54	–0.185	0.16
IL-5	0.13	0.63	–0.04	0.77
IL-6	0.65	0.006	0.26	0.046
IL-8	0.49	0.055	0.3	0.02
IL-10	0.25	0.36	–0.09	0.47
IL-12P70	–0.02	0.93	0.005	0.97
IL-17A	0.18	0.51	–0.2	0.14
TNF-α	0.44	0.09	0.25	0.053
IFN-α	0.24	0.38	–0.16	0.23
INF-γ	0.42	0.11	–0.01	0.91

### Transcriptomic Analysis of the Response of THP-1 Macrophages to Interleukin-26

**FIGURE 4:**
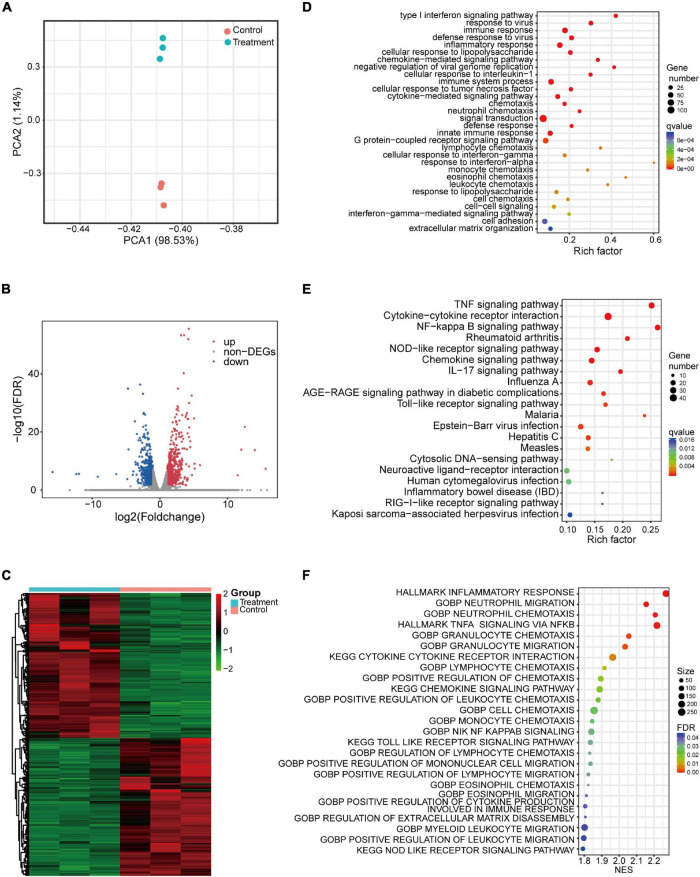
Transcriptomic analysis of THP-1 macrophages exposed to IL-26. **(A)** Principal component analysis of protein-coding genes. **(B)** Volcano plots showing protein-coding genes from untreated and IL-26-treated macrophages, red and blue indicate the significantly upregulated and downregulated genes, respectively. **(C)** Heatmap of differentially expressed protein-coding genes between untreated and IL-26-treated macrophages. **(D)** GO enrichment analysis (biological process) of up-regulated genes. **(E)** Top 20 from the KEGG enrichment analysis of up-regulated genes ranked by *q*-value. **(F)** GSEA analysis of all protein-coding genes in IL-26-treated macrophages.

Given the critical role of macrophages in IBD and the limited information on the immunoregulatory effects of IL-26 on macrophages, RNA-seq was used in the present study to identify the genomic changes in THP-1 macrophages induced by IL-26. Principal component analysis (PCA) showed that clusters of untreated macrophages were well-separated from those of IL-26-treated macrophages (Figure 4A). In total, we identified 1,303 differentially expressed protein-coding genes between untreated and IL-26-treated macrophages, including 667 up-regulated and 636 down-regulated genes (Figure 4B). The DEGs from biological replicates showed close clustering, indicating that the effects of IL-26 treatment were reproducible (Figure 4C).

GO enrichment analysis revealed the enrichment of up-regulated genes in the following biological processes: immune response, inflammatory response, chemokine-mediated signaling pathway, cytokine-mediated signaling pathway, chemotaxis, neutrophil chemotaxis, innate immune response, lymphocyte chemotaxis, monocyte chemotaxis, leukocyte chemotaxis, cell adhesion, and extracellular matrix organization (Figure 4D). Within the cellular component category, up-regulated genes were significantly enriched in plasma membrane, extracellular space, extracellular region, cell surface, and integral component of plasma membrane (Supplementary Figure 2A). Moreover, within the molecular function category, they were significantly enriched in cytokine activity and chemokine activity (Supplementary Figure 2A). Enrichment of down-regulated genes based on biological process is presented in Supplementary Figure 2B. In the KEGG pathway analysis, we noted that the up-regulated genes were significantly enriched in signaling pathways such as the TNF signaling pathway, cytokine-cytokine receptor interaction, NF-kappa B signaling pathway, NOD-like receptor signaling pathway, chemokine signaling pathway, IL-17 signaling pathway, toll-like receptor signaling pathway, and inflammatory bowel disease (IBD) (Figure 4E). Meanwhile, the down-regulated genes showed enrichment in gastric acid secretion.

Furthermore, in IL-26-exposed macrophages, GSEA of all protein-coding genes showed enrichment for the following: inflammatory response, neutrophil migration and chemotaxis, TNF-α signaling via NF-κB, cytokine-cytokine receptor interaction, lymphocyte chemotaxis, positive regulation of chemotaxis, chemokine signaling pathway, positive regulation of leukocyte chemotaxis, monocyte chemotaxis, toll-like receptor signaling pathway, positive regulation of mononuclear cell migration, positive regulation of cytokine production involved in immune response, regulation of extracellular matrix disassembly and NOD-like receptor signaling pathway (Figure 4F).

### Interleukin-26 Promotes Cytokine and Chemokine Secretion and the Expression of Adhesion Molecules in THP-1 Macrophages

**FIGURE 5:**
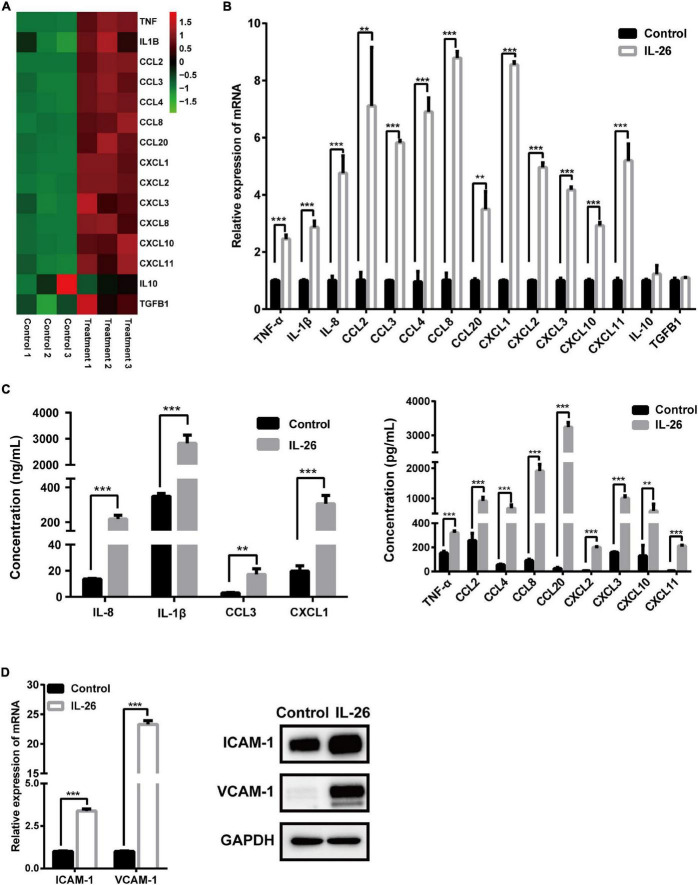
Effect of IL-26 exposure on mRNA and protein levels of cytokines, chemokines and adhesion molecules in THP-1 macrophages. **(A)** Heatmap of 15 immune- or inflammation-related genes. **(B)** mRNA levels of several cytokines and chemokines in THP-1 macrophages treated with IL-26. **(C)** Protein levels of several cytokines and chemokines in the supernatants from THP-1 macrophages exposed to IL-26. **(D)** mRNA and protein levels of ICAM-1 and VCAM-1 in THP-1 macrophages exposed to IL-26. Gene expression results were changed to Data are expressed as mean ± SD and are representative of three independent biological replicates. Images are representative of two independent biological replicates. **P < 0.01, ***P < 0.001.

To validate the results of RNA-seq analysis, the expression of 15 immune- or inflammation-related genes was evaluated using qRT-PCR and ELISA or western blots. Consistent with the results for these genes in RNA-seq analysis (Figure 5A), the mRNA levels of proinflammatory cytokines TNF-α, IL-1β, and IL-8 as well as chemokines CCL2, CCL3, CCL4, CCL8, CCL20, CXCL1, CXCL2, CXCL3, CXCL10, and CXCL11 were significantly elevated in macrophages in response to IL-26. In contrast, IL-10 and TGFB1 mRNA levels were unaffected by IL-26 (Figure 5B). Similarly, we observed increased protein secretion of TNF-α, IL-1β, IL-8, CCL2, CCL3, CCL4, CCL8, CCL20, CXCL1, CXCL2, CXCL3, CXCL10, and CXCL11 in conditioned media from macrophages treated with IL-26 (Figure 5C). In addition, IL-26 treatment also resulted in an elevation in the mRNA and protein levels of ICAM-1 and VCAM-1 in THP-1 macrophages (Figure 5D).

### Effect of Interleukin-26 Exposure on Matrix Metalloproteinase Expression in THP-1 Macrophages

**FIGURE 6:**
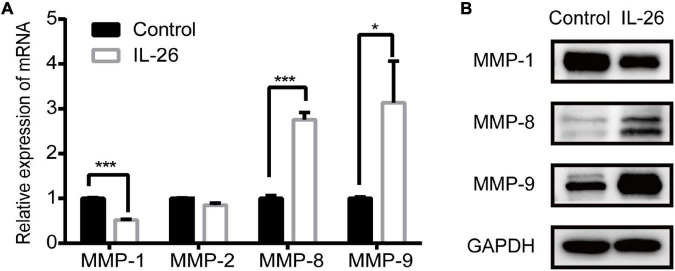
Effect of IL-26 on matrix metalloproteinases (MMPs) expression in THP-1 macrophages. **(A)** mRNA levels of MMP-1, MMP-2, MMP-8 and MMP-9 in THP-1 macrophages treated with IL-26. Results are expressed as mean ± SD and are representative of three independent biological replicates. **(B)** Protein expression of MMP-1, MMP-8 and MMP-9 in THP-1 macrophages treated with IL-26. Images are representative of two independent biological replicates. *P < 0.05, ***P < 0.001.

In the present study, GO enrichment analysis showed that up-regulated genes were significantly enriched in extracellular matrix organization (Figure 4D). Thus, we examined how IL-26 affects MMP expression in THP-1 macrophages. Results of qRT-PCR showed that IL-26 enhanced MMP-8 and MMP-9 levels and downregulated MMP-1 levels in THP-1 macrophages, whereas MMP-2 expression was unaffected (Figure 6A). Western blot analyses further confirmed that IL-26 upregulated MMP-8 and MMP-9 protein levels and reduced MMP-1 expression (Figure 6B).

## Discussion

In the present study, we observed that compared with HC, IBD patients showed elevated IL-26 levels in the inflamed intestinal mucosa, and reduced IL-26 levels in the peripheral blood. We provide the first evidence showing that IL-26 mRNA levels in PBMCs are associated with disease activity in IBD and disease behavior in CD. These results highlight the potential utility of circulating IL-26-producing cells in monitoring disease activity and risk stratification. Furthermore, to our knowledge, our study provides the first comprehensive analysis of transcriptomic changes in IL-26-exposed macrophages.

Surprisingly, we observed reductions in serum IL-26 levels in cases of IBD, irrespective of disease activity (Figures 2, 3), in contrast to findings from a previous German study reporting elevations in serum IL-26 levels in CD (11). This discrepancy may be the result of differences in genetic backgrounds and detection methods. Serum IL-22 levels have been found to be influenced by *IL23R* genotypes in CD (30). Both IL-22 and IL-26 belong to the IL-10 family and the *IL26* gene is located between the *IFNG* and *IL22* genes (31). Thus, we speculated that a similar phenomenon may be observed for IL-26. Moreover, we used ELISA for measuring serum IL-26 concentrations, while immunoluminometric assays were utilized in the previous study (11). Thus, serum IL-26 levels in IBD patients with different ethnic backgrounds need to be measured using the same method to elucidate this difference. Furthermore, serum IL-26 levels were found to be positively correlated with serum IL-6 and IL-8 levels in CD patients and with serum IL-6 levels in UC patients in our study (Table 1). This was consistent with a study that found a positive correlation between IL-26 and IL-8 levels in cell-free bronchoalveolar lavage (BAL) fluid from patients with asthma, although these patients showed lower IL-26 concentrations in BAL fluid than did controls (32).

The link between blood and gut IL-26 remains unclear. Our study showed that IL-26 mRNA and protein levels are higher in the inflamed intestinal mucosa of IBD patients than in paired uninflamed intestinal mucosa and HC (Figure 1), confirming and extending the findings of previous studies (5, 11). However, IL-26 expression levels in serum and PBMCs were lower in IBD patients than in HC (Figure 2). Interestingly, IL-26 mRNA levels in PBMCs appeared to be negatively correlated with disease activity in IBD (Figure 3). Thus, we speculate that in IBD, the migration of IL-26-producing PBMCs into the inflamed intestine may result in reduced expression of IL-26 mRNA in PBMCs and elevated expression of IL-26 in the inflamed intestinal mucosa. Another possible explanation is that gut and blood IL-26 may play distinct roles in the etiology of IBD. IL-26 not only acts as a cytokine but also mediates the antimicrobial response against extracellular and intracellular bacteria (31, 33). When bacterial DNA (bactDNA) translocates into the blood, serum IL-26 levels rise in CD patients in remission, and this upregulation is compromised in patients with the variant IL-26 (varIL26) genotype. In the presence of bactDNA and infliximab, PBMCs from varIL26-genotype CD patients show lower bacteria-killing ability, and higher pro-inflammatory cytokine secretion and anti-TNF-α antibody consumption than do those with the wild-type IL-26 genotype. Moreover, this phenomenon can be partially reversed by IL-26 supplementation (34). Therefore, blood IL-26 may be protective, helping in the clearance of bactDNA from the circulation, preventing a sustained inflammatory environment, and further reducing anti-TNF-α consumption, which suggest that circulating IL-26 levels may be associated with early biologic therapy and their therapeutic response. However, IL-26 can promote the secretion of IL-6 and IL-8 from human SEMFs and the expression of TNF-α and IL-8 in human intestinal epithelial cells (5, 11), indicating that it has a pro-inflammatory role in local intestinal inflammation. Together, the migration of IL-26-expressing PBMCs into the intestinal mucosa or the different biological function of blood and gut IL-26 in IBD may explain this phenomenon, although further exploration via experimental studies is warranted.

Apart from the aforementioned cell migration, host-microbe interactions may also contribute to elevated IL-26 levels in inflamed intestinal mucosa of IBD patients. Previous studies have shown that administration of the gut microbiota from IBD patients can promote the accumulation of Th17 cells and inflammatory macrophages, the cellular sources of IL-26, in the mouse gut (35, 36). Additionally, the IL-19 production in macrophages and IL-22 production in intestinal Th17 cells can be enhanced by the bacterial products LPS and butyrate, respectively (37, 38). Thus, we speculate that in the intestinal mucosa of IBD, IL-26 expression may be induced by the microbiota and microbial products. Previous findings showing that IL-26 levels are obviously elevated in BAL fluid from HC after intra-bronchial exposure to endotoxin are compatible with our hypothesis (17), since the respiratory and gastrointestinal tracts are both continuously exposed to external antigens, sharing similar structures.

Inflammatory macrophages in the intestinal mucosa can recruit and activate other immune cells by secreting several inflammatory mediators, thereby exacerbating the dysregulated intestinal immune response. In the present study, we identified global transcriptional alterations in IL-26-treated macrophages (Figure 4), extending previous findings (24). Both GSEA and GO enrichment analysis of up-regulated genes showed that neutrophil, monocyte, and lymphocyte chemotaxis was significantly enriched. KEGG enrichment analysis revealed that the up-regulated genes were significantly enriched in inflammation-related pathways, including TNF signaling pathway, cytokine-cytokine receptor interaction, NF-κB signaling pathway, chemokine signaling pathway, NOD-like and toll-like receptor signaling pathways, and inflammatory bowel disease (IBD). These findings suggest that IL-26 can activate macrophages and thereby trigger subsequent inflammatory and immune responses, pointing to a potential mechanism of IL-26 involvement in IBD pathology.

Increasing evidence suggests that excessive IL-26-induced neutrophil infiltration into the area of inflammation is detrimental to mice with psoriasis-like skin lesion and sepsis (14, 16). We found that IL-26 promotes the release of the neutrophil chemoattractants TNF-α, IL-1β, IL-8, CXCL1, CXCL2, CXCL3, and CCL3 from THP-1 macrophages. These cytokines may promote neutrophil infiltration into intestinal tissue and thereby aggravate intestinal inflammation. Moreover, we found that IL-26 also enhances the release of CXCL11 and CCL20 from macrophages. Conversely, IL-1β can promote the secretion of IL-26 from Th17 cells (39). CXCL11 promotes Th17 cell development, and CCL20 facilitates the migration of Th17 cells to inflammatory sites (40, 41). This cytokine induction loop between macrophages and Th17 cells may drive the ongoing inflammation. Additionally, in this study, IL-26 was observed to enhance the release of CCL2, CCL8, and CXCL10 and the expression of the adhesion molecules ICAM-1 and VCAM-1 in macrophages. CCL2, CCL8, and CXCL10 have been described to recruit inflammatory monocytes and macrophages or Th1 cells to promote colitis, respectively (42–45). ICAM-1 is considered a marker of macrophage activation (46). Increased VCAM-1 expression in macrophages during chronic inflammation is suggested to mediate antigen presentation to infiltrating lymphocytes (47). These results highlight the possibility that IL-26 may act as an upstream signal that promotes macrophage activation and subsequently recruits neutrophils, monocytes, macrophages, and T cells to the intestinal tissue, thus contributing to intestinal inflammation. In contrast, it has been reported that IL-26 could protect mice against acute DSS-induced colitis, which is accompanied by reduced expression of TNF, CXCL9, and CXCL10, although its precise role in chronic colitis remains unclear (12). Taken together, IL-26 seems to play multifaced roles in the different phase of colitis, and its contribution to IBD should be studied further.

The findings of this study suggest that IL-26 may participate in ECM remodeling in IBD by regulating MMP expression in macrophages. GO enrichment analysis of up-regulated genes revealed enrichment in extracellular matrix organization. Moreover, GSEA showed that a similar biological process was also found to be enriched in IL-26-treated macrophages (Figures 4D,F). We further found that IL-26 can promote the mRNA and protein expression of MMP-8 and MMP-9 while inhibiting the expression of MMP-1 (Figure 6). The overexpression of MMP-1, MMP-8, and MMP-9 is observed in the intestinal mucosa of IBD patients (48). MMP-9 has been implicated in the breakdown of intestinal epithelial barriers, and infiltrating macrophages are its primary producers in IBD (49, 50). MMP-9 in inflammatory macrophages promotes macrophage migration across the ECM during the inflammatory response (51). Additionally, MMP-8 in macrophages also allows the migration of macrophages across type I collagen (52, 53). Thus, the upregulation of MMP-8 and MMP-9 in macrophages may lead to increased macrophage infiltration in the inflamed intestinal tissue.

There are some questions that remain unanswered and warrant investigation in the future. We showed that IBD patients exhibit increased IL-26 expression in the inflamed intestinal mucosa, but reduced IL-26 levels in serum and PBMCs compared with HC. The possible mechanisms underlying this phenomenon, such as the migration of circulating IL-26 expressing cells or distinct roles of IL-26 in the intestine and peripheral blood, need to be explored further. Interestingly, we found that IL-26 mRNA levels in PBMCs were associated with disease activity in IBD and linked to complicated CD. However, we could not compare the IL-26 levels in serum and PBMCs among UC patients with different disease locations due to the limited sample size. Thus, more UC patients are required to perform this comparison. Additionally, the IL-26 protein expression in different subpopulation of PBMCs was not investigated using flow cytometry. It is worthwhile to further investigate which unique subset of circulating IL-26-producing cells is associated with these clinical features. Importantly, longitudinal studies are required to explore the possible value of circulating IL-26-expressing cells and IL-26 levels in clinical applications such as prognostication and personalized treatment. Lastly, although we explored the immunoregulatory effects of IL-26 on macrophages *in vitro*, further research is warranted to delineate the role of IL-26 in different phase of colitis *in vivo* using human IL-26 transgenic mice.

Some previous studies have demonstrated interactions among IL-10 cytokine family members. IL-10, IL-19, IL-20, IL-22, and IL-24 have also been implicated in the etiology of IBD (54–56). IL-26 was reported to enhance the mRNA expression of IL-19, IL-20, and IL-24 in monocytes and increase IL-22 production in memory T cells (6). IL-26 has also been described to promote and inhibit IL-10 secretion in mouse macrophages and human CD4^+^ T cells cocultured with autologous monocytes, respectively (7, 23). Moreover, IL-10 was found to attenuate IL-26-induced IL-6 secretion in monocytes (6). Additionally, IL-19 and IL-22 were reported to produce synergistic effects on human colonic SEMFs, despite not sharing the same receptor chain (55). Thus, further studies are required to elucidate the potential interactions between IL-26 and other IL-10 family members under conditions of chronic intestinal inflammation, contributing to a better understanding of the complex cytokine network involved in IBD.

## Conclusion

To conclude, we observed that IL-26 levels were elevated in the inflamed intestinal mucosa, but reduced in serum and PBMCs of IBD patients as compared with HC. Importantly, to our knowledge, we showed for the first time that IL-26 mRNA levels in PBMCs are negatively correlated with disease activity in both UC and CD and are downregulated in patients with complicated CD, but no such association can be observed for serum IL-26 levels. Additionally, the transcriptomic changes in IL-26-exposed macrophages highlight that IL-26 may participate in the progression of IBD via the activation of macrophages. The findings in the present study suggest that IL-26 levels in PBMCs may serve as a marker of disease activity, and IL-26 may be a novel therapeutic target for IBD.

## Data Availability Statement

The datasets presented in this study can be found in online repositories. The names of the repository/repositories and accession number(s) can be found below: GEO, GSE192493.

## Ethics Statement

The studies involving human participants were reviewed and approved by the Medical Ethics Committee of Renji Hospital, School of Medicine, Shanghai Jiao Tong University. The patients/participants provided their written informed consent to participate in this study.

## Author Contributions

DS performed the experiments, conducted the data analysis, and wrote the manuscript. LL collected the data. JL and JT provided the clinical specimen. ZR contributed to study design and critical revision of the manuscript. All authors contributed to the article and approved the submitted version.

## Conflict of Interest

The authors declare that the research was conducted in the absence of any commercial or financial relationships that could be construed as a potential conflict of interest.

## Publisher’s Note

All claims expressed in this article are solely those of the authors and do not necessarily represent those of their affiliated organizations, or those of the publisher, the editors and the reviewers. Any product that may be evaluated in this article, or claim that may be made by its manufacturer, is not guaranteed or endorsed by the publisher.

## Footnotes

1
https://www.gsea-msigdb.org/gsea/msigdb/index.jsp
