# Supplementary material for: Interleukin-26 Expression in Inflammatory Bowel Disease and Its Immunoregulatory Effects on Macrophages
Source: Front Med (Lausanne). 2022 Apr 6;9:797135. doi: 10.3389/fmed.2022.797135 (PMC9019154; doi:10.3389/fmed.2022.797135)
Supplement: Supplementary file 1 [file Data_Sheet_1.docx]

Supplementary Table 1. Demographic and clinical characteristics of the study population at intestinal biopsy.

|  | **Control (n = 45)** | **UC (n = 37)** | **CD (n = 26)** |
| --- | --- | --- | --- |
| Male, n (%) | 29 (64.44%) | 20 (54.05%) | 17 (65.38%) |
| Age, y, median (IQR) | 37 (31-41) | 35 (31-50.5) | 31.5 (26.75-36) |
| **Disease location, n (%)** |  |  |  |
| L1 ± L4 | / |  | 8 (30.77%) |
| L2 ± L4 | / |  | 1 (3.85%) |
| L3 ± L4 | / |  | 17 (65.38%) |
| E1 (Proctitis) | / | 7 (18.92%) |  |
| E2 (Left-sided colitis) | / | 24 (64.86%) |  |
| E3 (Pancolitis) | / | 6 (16.22%) |  |
| **Disease behavior, n (%)** |  |  |  |
| B1 (Non‐stricturing,  non‐penetrating) | / |  | 13 (50%) |
| B2 (Stricturing) | / |  | 13 (50%) |
| B3 (Penetrating) | / |  | 0 |
| **Histological activity**  (normal/quiescent/active) | / | 1/4/26^*^ | 0/8/18 |
| **Endoscopic activity**  (remission/active) | / | 2/35 | 4/22 |

y, years; IQR, interquartile range.

^*^Pathological reports were not available for six UC patients.

Supplementary Table 2. Demographic and clinical characteristics of the study population included in IHC analysis.

|  | **Control (n = 23)** | **UC (n = 22)** | **CD (n = 28)** |
| --- | --- | --- | --- |
| Male, n (%) | 16 (69.57%) | 8 (36.36%) | 17 (60.71%) |
| Age, y, median (IQR) | 38 (32-45) | 37.5 (29-55) | 31.5 (29-39.75) |
| **Disease location, n (%)** |  |  |  |
| L1 ± L4 | / |  | 9 (32.14%) |
| L2 ± L4 | / |  | 1 (3.57%) |
| L3 ± L4 | / |  | 17 (60.71%) |
| L4 (isolated upper gastrointestinal disease) |  |  | 1 (3.57%) |
| E1 (Proctitis) | / | 3 (13.64%) |  |
| E2 (Left-sided colitis) | / | 13 (59.09%) |  |
| E3 (Pancolitis) | / | 6 (27.27%) |  |
| **Disease behavior, n (%)** |  |  |  |
| B1 (Non‐stricturing,  non‐penetrating) | / |  | 11 (39.29%) |
| B2 (Stricturing) | / |  | 17 (60.71%) |
| B3 (Penetrating) | / |  | 0 |
| **Histological activity**  (normal/quiescent/active) | / | 0/2/20 | 0/7/21 |
| **Endoscopic activity**  (remission/active) | / | 2/20 | 2/26 |

y, years; IQR, interquartile range.

Supplementary Table 3. Demographic and clinical characteristics of the study population included in blood IL-26 analysis.

|  | **Control (n= 54)** | **UC (n= 18)** | **CD (n= 61)** |
| --- | --- | --- | --- |
| Male, n (%) | 33 (61.1%) | 9 (50%) | 49 (80.3%) |
| Age, y, median (IQR) | 34 (27.75-45.25) | 40 (30.5-55.25) | 28 (23-38) |
| **Disease location, n (%)** |  |  |  |
| L1 ± L4 | / | / | 21 (34.43%) |
| L2 ± L4 | / | / | 3 (4.92%) |
| L3 ± L4 | / | / | 37 (60.65%) |
| E1 (Proctitis) | / | 1 (5.55%) | / |
| E2 (Left-sided colitis) | / | 3 (16.67%) | / |
| E3 (Pancolitis) | / | 14 (77.78%) | / |
| **Disease behavior, n (%)** |  |  |  |
| B1 (Non-stricturing, non-penetrating) | / | / | 37 (60.65%) |
| B2 (Stricturing)^*^ | / | / | 23 (37.7%) |
| B3 (Penetrating) | / | / | 2 (3.28%) |
| **Disease activity, n** |  |  |  |
| remission/mild/moderate/severe | / | 0/6/4/8 | 9/37/15/0 |
| **Laboratory tests, median (IQR)** |  |  |  |
| WBC count, 10^9^/L | 5.49 (4.34-6.33) | 6.99 (5.74-8.79) | 6.05 (4.88-7.33) |
| Hb, g/L | 146.5 (133-153.25) | 120 (98.75-137.5) | 137 (121.5-144) |
| CRP, mg/L | / | 10.45 (0.5-37.94) | 2.59 (0.5-11.84) |
| ESR, mm/h | / | 37 (6.75-55.25) | 10 (6-23) |
| PA, mg/L | 272.95 (250.43-303.25) | 183.15 (127.85-245.93) | 225.2 (187.15-273) |
| **Treatment, n** |  |  |  |
| Aminosalicylates | / | 14 | 10 |
| Steroids | / | 4 | 3 |
| IMM | / | 2 | 10 |
| Biologics | / | 4 | 28 |
| None | / | 0 | 9 |
| Others | / | 1 | 9 |

y, years; IQR, interquartile range; WBC, white blood cells; Hb, hemoglobin; CRP, C-reaction protein; ESR, erythrocyte sedimentation rate; PA, prealbumin. IMM, immunomodulators.

^*^1 CD patients was identified to have both stricturing and penetrating phenotype.

Supplementary Table 4. Primers used for quantitative real time PCR.

| **Target gene** | **Forward primer sequences** | **Reverse primer sequences** |
| --- | --- | --- |
| IL-26 | GCTGTTAGTCACTCTGTCTCTTG | GGACAATGTTCCCCTTGGGTA |
| TNF-α | CTCTTCTGCCTGCTGCACTTTG | ATGGGCTACAGGCTTGTCACTC |
| IL-1β | ATGATGGCTTATTACAGTGGCAA | GTCGGAGATTCGTAGCTGGA |
| IL-8 | GAGAGTGATTGAGAGTGGACCAC | CACAACCCTCTGCACCCAGTTT |
| IL-10 | TCTCCGAGATGCCTTCAGCAGA | TCAGACAAGGCTTGGCAACCCA |
| TGFB1 | TACCTGAACCCGTGTTGCTCTC | GTTGCTGAGGTATCGCCAGGAA |
| CCL2 | CAGCCAGATGCAATCAATGCC | TGGAATCCTGAACCCACTTCT |
| CCL3 | AGTTCTCTGCATCACTTGCTG | CGGCTTCGCTTGGTTAGGAA |
| CCL4 | CTCCCAGCCAGCTGTGGTATTC | CCAGGATTCACTGGGATCAGCA |
| CCL8 | TATCCAGAGGCTGGAGAGCTAC | TGGAATCCCTGACCCATCTCTC |
| CCL20 | TGCTGTACCAAGAGTTTGCTC | CGCACACAGACAACTTTTTCTTT |
| CXCL1 | AGCTTGCCTCAATCCTGCATCC | TCCTTCAGGAACAGCCACCAGT |
| CXCL2 | GGCAGAAAGCTTGTCTCAACCC | CTCCTTCAGGAACAGCCACCAA |
| CXCL3 | CCAAACCGAAGTCATAGCCAC | TGCTCCCCTTGTTCAGTATCT |
| CXCL10 | GGTGAGAAGAGATGTCTGAATCC | GTCCATCCTTGGAAGCACTGCA |
| CXCL11 | GACGCTGTCTTTGCATAGGC | GGATTTAGGCATCGTTGTCCTTT |
| ICAM-1 | GTATGAACTGAGCAATGTGCAAG | GTTCCACCCGTTCTGGAGTC |
| VCAM-1 | GGGAAGATGGTCGTGATCCTT | TCTGGGGTGGTCTCGATTTTA |
| MMP-1 | GGGGCTTTGATGTACCCTAGC | TGTCACACGCTTTTGGGGTTT |
| MMP-2 | CCCACTGCGGTTTTCTCGAAT | CAAAGGGGTATCCATCGCCAT |
| MMP-8 | CAACCTACTGGACCAAGCACAC | TGTAGCTGAGGATGCCTTCTCC |
| MMP-9 | AGACCTGGGCAGATTCCAAAC | CGGCAAGTCTTCCGAGTAGT |
| ACTB | AGAGCCTCGCCTTTGCCGATCC | CTGGGCCTCGTCGCCCACATA |
| GAPDH | GTCTCCTCTGACTTCAACAGCG | ACCACCCTGTTGCTGTAGCCAA |





Supplementary Figure 1. IL-26 expression levels in serum and PBMCs in CD patients according to disease location and behavior.

(A and B) IL-26 levels in serum (A) and PBMCs (B) in CD patients with terminal ileum involvement (L1±L4) and colonic involvement (L2/L3±L4). (C and D) IL-26 levels in serum (C) and PBMCs (D) in CD patients with and without upper gastrointestinal involvement. (E and F) IL-26 levels in serum (E) and PBMCs (F) in complicated CD. ^**^*P* < 0.01.


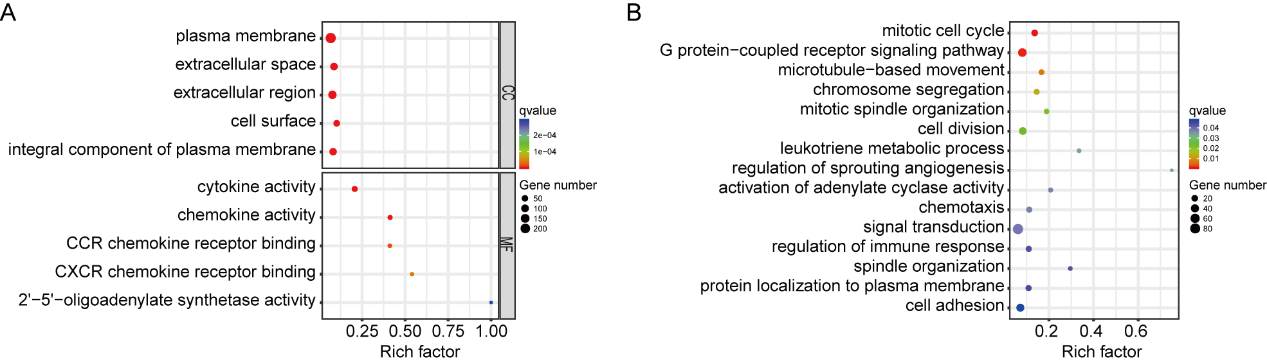


Supplementary Figure 2. Functional enrichment analysis of differentially expressed protein-coding genes.

(A) Top 5 GO enrichment terms for up-regulated genes, categorized by molecular function (MF), and cellular component (CC), ranked by q-value. (B) GO enrichment analysis (biological process) of down-regulated genes.
